# Diffuse large B cell lymphoma presenting as Horner's syndrome in a patient diagnosed with neurofibromatosis type 1: a case report and review of the literature

**DOI:** 10.1186/1752-1947-6-8

**Published:** 2012-01-11

**Authors:** Saoraya Lueangarun, Chirayu U Auewarakul

**Affiliations:** 1 Department of Medicine, Faculty of Medicine Siriraj Hospital, Mahidol University, 2 Prannok Road, Bangkok 10700, Thailand; 2 Division of Hematology, Department of Medicine, Faculty of Medicine Siriraj Hospital, Mahidol University, 2 Prannok Road, Bangkok 10700, Thailand

## Abstract

**Introduction:**

Horner's syndrome has a variety of etiologies ranging from benign to serious life-threatening conditions and has been infrequently reported as a presenting symptom of patients with lymphoid neoplasms. Only one case of Burkitt's lymphoma presenting with toothache, paresthesia, and Horner's syndrome has been described and no case reports of diffuse large B-cell lymphoma as the etiology of Horner's syndrome currently exist in the literature. In addition, lymphoid neoplasms have rarely been reported to occur in patients with neurofibromatosis type 1 despite an increased risk of many types of cancer in such cases.

**Case presentation:**

A 28-year-old Thai man presented with a progressively enlarged left supraclavicular mass together with a significant weight loss and night sweating for four months. He also noticed hoarseness and ptosis of his left eye associated with double vision for two months. Physical examination revealed large supraclavicular lymphadenopathy and Horner's syndrome (ptosis, miosis, and anhydrosis) on the left side of his face. A large mediastinal mass was clearly detected by chest X-ray and computed tomography and subsequent lymph node biopsy provided a diagnosis of diffuse large B-cell lymphoma. Interestingly, the patient was also definitely diagnosed with neurofibromatosis type 1 from multiple café au lait macules, axillary freckles, three neurofibromas, multiple Lisch nodules, and a history of affected family members. He subsequently received chemotherapy with a good response. Twenty-seven cases of various types of lymphoid neoplasms previously reported to occur in neurofibromatosis type 1 patients were also extracted from the literature. All cases were non-Hodgkin lymphoma and the major subtype was T-cell. Only nine cases were B-cell lymphoma. The majority of cases were young with a median age at lymphoma diagnosis of 9.4 years (range 1.1 to 77 years). Two-thirds of the cases were boys or men. Other concomitant malignancies were brain tumor, colorectal cancer, pheochromocytoma, and acute lymphoblastic leukemia.

**Conclusions:**

We describe for the first time a case of diffuse large B-cell lymphoma that occurred in a neurofibromatosis type 1 patient with Horner's syndrome. Horner's syndrome can be an initial manifestation of diffuse large B-cell lymphoma. Patients who present with a classical triad of Horner's syndrome should always be fully investigated for lymphomatous involvement, especially in the thorax. The exact molecular mechanism for diffuse large B-cell lymphoma development in neurofibromatosis type 1 cases remains to be elucidated.

## Introduction

Neurofibromatosis type 1 (NF1) (formerly known as von Recklinghausen disease) is an autosomal dominant neurocutaneous disorder characterized by several distinct clinical features such as café au lait macules, intertriginous freckling, Lisch nodules, neurofibromas, osseous dysplasia, and a family history of first-degree relatives affected by NF1 [1-3]. A variety of neoplasms have been reported to occur in association with NF1 including optic pathway gliomas, astrocytomas, brainstem gliomas, and malignant peripheral nerve sheath tumors (MPNSTs) [4-6]. Other types of tumors were also described such as pheochromocytoma, chronic myeloid leukemia, rhabdomyosarcoma, and gastrointestinal stromal tumors [1,7-9]. However, the existence of lymphoid neoplasms in patients with NF1, particularly diffuse large B-cell lymphoma (DLBCL), has been infrequently reported [1,9].

Horner's syndrome is a recognized neurological syndrome consisting of ptosis, pupillary miosis, and facial anhydrosis [10]. The syndrome occurs as a result of the interruption of the oculosympathetic pathway which could occur along its route from the hypothalamus, brain stem, spinal cord, brachial plexus, lung apex, carotid artery, cavernous sinus and finally to the eye [10,11]. Three types of Horner's syndrome exist according to the anatomical level of the defect, that is, central, preganglionic, and postganglionic [12]. A variety of disorders have been described in association with Horner's syndrome ranging from brain stem ischemia, brain tumors, demyelinating diseases, direct spinal cord trauma, iatrogenic disruption of the sympathetic pathway from radical neck dissection, carotid angiography, stenting or endarterectomy, spontaneous carotid dissection, aortic aneurysm to various malignant conditions that directly or indirectly affect the normal sympathetic innervations [10-12]. Primary and metastatic lung carcinoma, Pancoast tumor, thyroid carcinoma, neuroblastoma, Burkitt's lymphoma, and Hodgkin's disease have all been described as the causes of Horner's syndrome [10-13]. Interestingly, DLBCL which is the most common hematologic malignancy worldwide has never been shown to be associated with Horner's syndrome at the outset. In addition, the occurrence of DLBCL as the etiology of Horner's syndrome in an NF1 patient has not been described in the literature. We describe for the first time the case of a young Thai man with NF1 who was confirmed by pathology as having DLBCL after he presented with a classical Horner's syndrome.

## Case presentation

A 28-year-old Thai man presented to Siriraj Hospital with a history of a progressively enlarged left supraclavicular mass, significant weight loss, and night sweats for four months.

**Figure 1:**
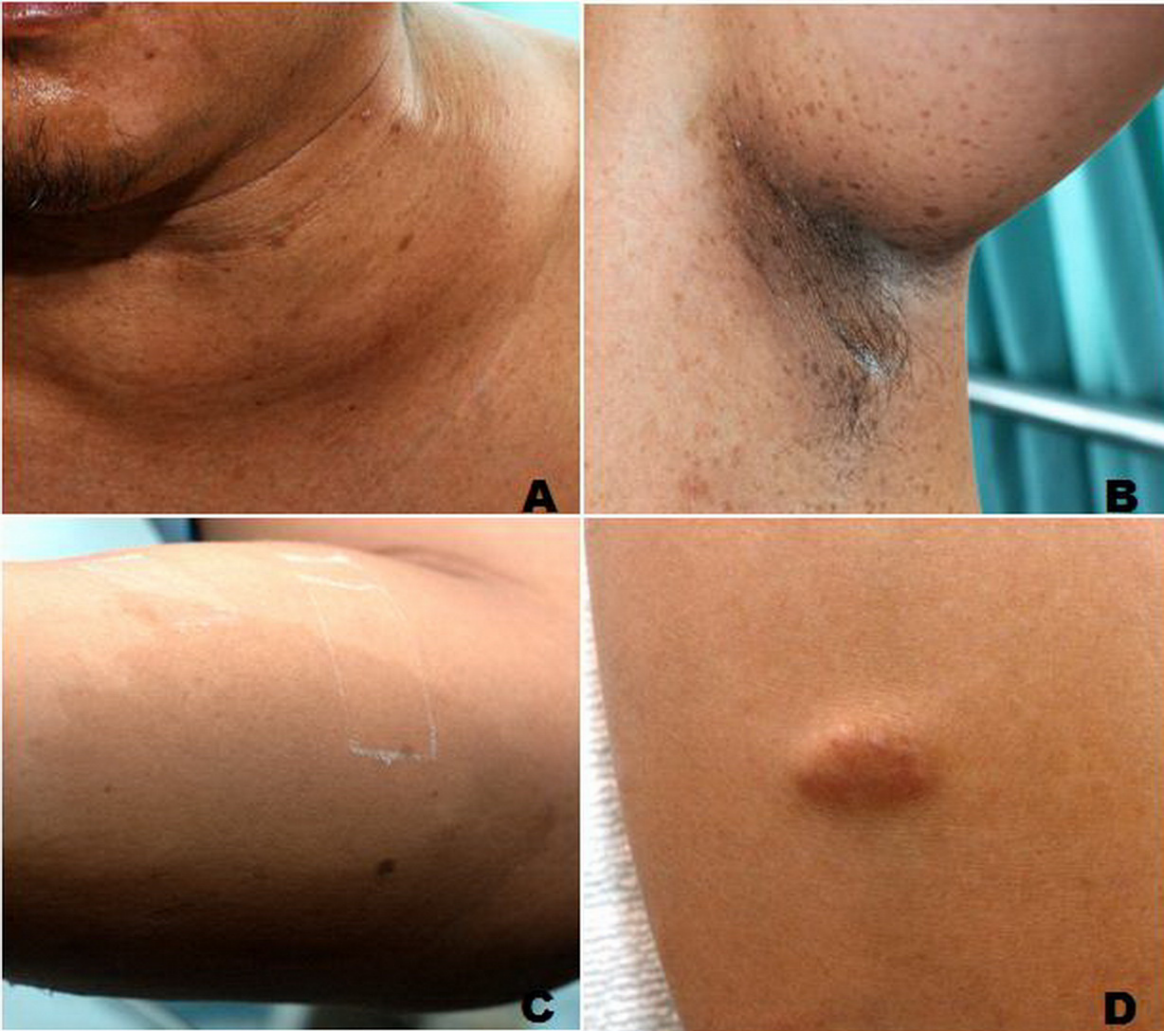
**The patient's clinical presentations**;neck mass (A), axillary freckles (B), café-au-lait macules (C), and neurofibroma (D).

Two months prior to this admission, he noticed hoarseness associated with ptosis of his left eye and double vision. The physical examination at presentation revealed ptosis of his left eye with a miotic pupil and anhydrosis on his left hemifacial area, all of which were compatible with Horner's syndrome. His skin examination disclosed several dermatologic signs such as multiple well-defined brownish café au lait patches on the abdomen, areolae, arms, back and buttocks, and axillary freckles (Figure 1). There were also soft movable skin-colored nodules located on his left thigh, left forearm, and back. Subsequent ophthalmologic slit-lamp examination detected multiple Lisch nodules which are raised pigmented hamartomas of the iris. He reported having multiple pigmented skin lesions and rubbery nodular skin lesions over his entire body since childhood. His father also had similar skin lesions over the whole body area. Other pertinent examination included a large non-tender and rubbery mass (15 cm in length and 10 cm in width) in the left supraclavicular region with no palpable lymph nodes elsewhere in the body.

**Figure 2:**
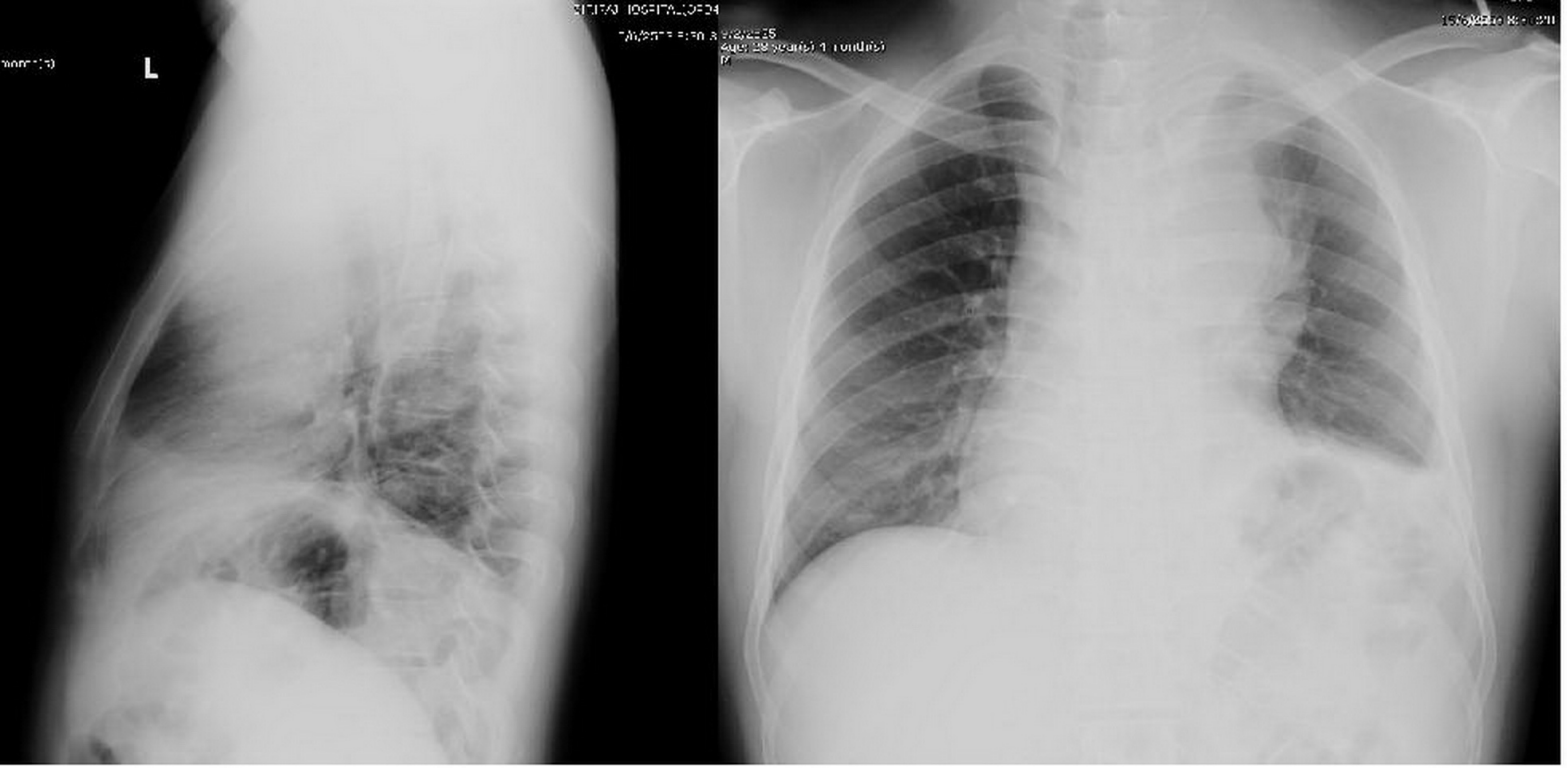
**Chest X-ray revealed a large anterior mediastinal mass**.

**Figure 3:**
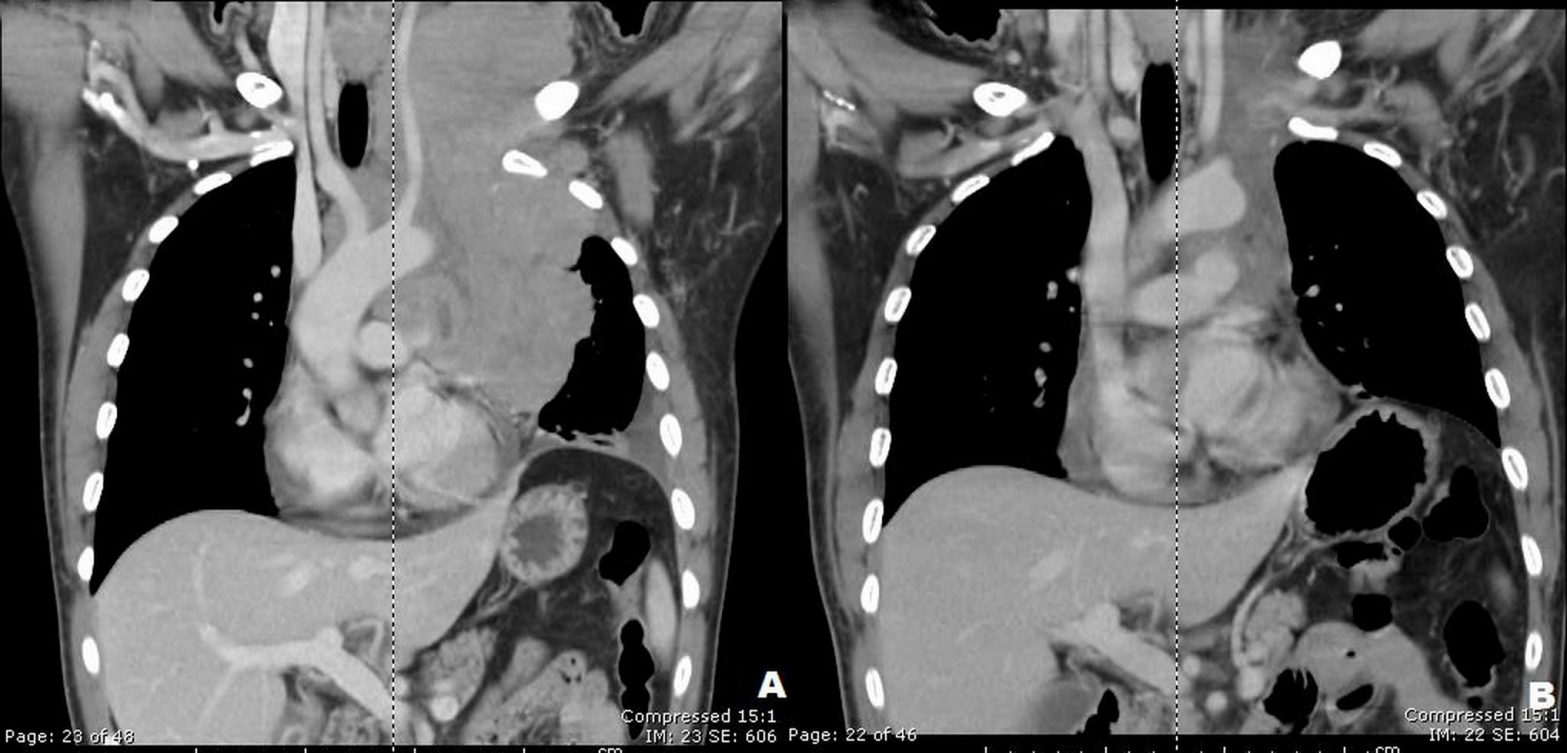
**Computed tomography (CT) of the neck and chest showed a large lobulated heterogeneous-enhancing mass with central necrosis at the anterior and the middle mediastinum with some parts extending into the left anterior neck** (A). Significant improvement of lymphomatous involvement at the prevascular region was seen after eight cycles of CHOP chemotherapy (B). CHOP, Cyclophosphamide (Cytoxan), Hydroxyrubicin (Adriamycin), Oncovin (Vincristine), Prednisone.

Routine laboratory tests revealed a hemoglobin level of 14.5 g⁄dL, a total leukocyte count of 8.2 × 10^9^/L (70% neutrophils, 22% lymphocytes, and 8% monocytes) and a platelet count of 280 × 10^9^/L. Serum lactate dehydrogenase (LDH) was 525 U/L (normal range, 225 to 450 U/L) and serum uric acid was 13.4 mg/dL (normal range, 2.4 to 7 mg/dL). His liver function tests were normal. Chest X-ray revealed a large anterior mediastinal mass (Figure 2). Computed tomography (CT) of his neck showed matted lymphadenopathy on the left side of the neck, which extended along the carotid vessels to the left thoracic cavity. Computed tomography (CT) of the chest disclosed a large (10.9 × 9.7 × 18 cm) lobulated heterogeneous-enhancing mass with a central necrosis in the anterior and middle mediastinum which extended superiorly into the left anterior neck (C5-C6 level), and encased around his aortic arch, left subclavian artery, left jugular vein, trachea, left main bronchus and left pulmonary artery (Figure 3A). Multiple subcentimeter mediastinal lymphadenopathies were also observed. Left pleural effusions with adjacent atelectasis of the left lower lobe were also present. From these findings, the differential diagnoses of this mass were lymphoma, teratoma, lung cancer or metastasis and malignancy associated with NF1, such as MPNSTs and chromaffin cells tumor. Therefore, supraclavicular lymph node biopsy was performed to make a definite pathological diagnosis which revealed diffuse, mixed small and large lymphoid cells compatible with malignant lymphoma (intermediate grade). Immunostaining of the cells demonstrated that neoplastic cells were marked with CD20, CD10, CD43, BCL2, BCL6, and MUM1, but not with CD3, CD5, CD23, CD34, TdT, or cyclin D1. Kappa but not lambda light chain restriction was also demonstrated. The malignant cells possessed a B-cell phenotype with mixed germinal center B-cell and activated B-cell features which were consistent with a DLBCL subtype according to the 2008 World Health Organization (WHO) Classification of neoplasms of the hematopoietic and lymphoid tissues [14]. Staging studies showed no bone marrow involvement and computed tomography (CT) of the whole abdomen revealed normal attenuation of liver parenchyma without a definite space occupying lesion. The spleen was unremarkable and no intraabdominal lymphadenopathy could be demonstrated. Once the diagnosis and the staging were completed, he was treated with the standard CHOP (Cyclophosphamide (Cytoxan), Hydroxyrubicin (Adriamycin), Oncovin (Vincristine), Prednisone) chemotherapy regimen for eight cycles, to which the tumor responded well (Figure 3B).

## Discussion

This report describes the case of an NF1 patient who presented with a rare manifestation of malignant lymphoma, that is, Horner's syndrome. In our case, NF1 could be definitely diagnosed according to the National Institute of Health Consensus Development Conference Diagnostic Criteria which require the presence of six or more café au lait macules, two or more subcutaneous neurofibromas, axillary or groin freckling, and two or more Lisch nodules seen on slit lamp examination [2]. An autosomal dominant family history was also demonstrated in this patient which goes along well with the typical NF1 inheritance pattern. Although it is well-known that NF1 patients have an increased risk of malignancies with an estimated risk of 5% to 15% in affected individuals, most reported types of malignancies are not lymphoid neoplasms [8,15]. Since the association between NF1 and malignant lymphoma is not fully recognized, we searched the literature through the US National Library of Medicine (PUBMED) using the keywords "malignant lymphoma", "lymphoma", and "neurofibromatosis" to find out if malignant lymphoma had been previously reported in NF1 cases and how frequently they had appeared in the literature. Only 27 cases of malignant lymphoma were reported in NF1 patients [7,16-34], and only two of them were DLBCL [27,33]. Table 1 summarizes details of all the reported cases except one case that was published in the non-English literature [35].

**Table 1:** Summarized data of all reported NF1 cases with lymphoma development

Case **No**.	References	Sex/ Age at onset (y)	Lymphoma	Other tumor(s)	Presentation of lymphoma	Family history of NF1	Family history of cancer	NF criteria*	Treatment	Outcome
1.	[16]	F, 20	Burkitt lymphoma	Pheochromocytoma	dental extract site mass	NA	NA	CALM, neurofibromas	Chemotherapy and radiotherapy	Alive

2.	[17]	M, 11	Diffuse, poorly differentiated lymphoma	Glioblastoma multiforme	anterior mediastinal mass	Y	Y	CALM	Chemotherapy	Death

3.	[18]	F, 6	T-cell lymphoblastic lymphoma	Gardner syndrome	mediastinal mass and pleural effusion	Y	Y	CALM	Chemotherapy and radiotherapy	Death

4.	[18]	F, 1.1	T-cell lymphoblastic lymphoma		neck mass	Y	Y	CALM	Radiotherapy	Death

5.	[18]	M, 4	T-cell lymphoblastic lymphoma		mediastinal mass and pleural effusion	Y	Y	CALM, neurofibromas	Chemotherapy and radiotherapy	Death

6.	[7]	M, 16	T-cell lymphoma	Acute lymphoblastic leukemia,15 months after diagnosis of lymphoma	mediastinal mass	Y	Y	CALM	Chemotherapy and radiotherapy	Death

7.	[19]	M, 45	Well-differentiated lymphocytic lymphoma	Pheochromocytoma, renal artery stenosis	abdominal mass	NA	NA	CALM, neurofibromas	NA	Alive

8.	[20]	M, 59	Diffuse, medium sized cell type, B cell type NHL		lymphadenopathy	Y	NA	CALM, neurofibromas	Chemotherapy	Death

9.	[21]	M, 7.8	T-NHL	Acute lymphoblastic leukemia, prior to NHL		Y	Y	NA	NA	Death

10.	[21]	F, 4.1	T-NHL			Y	Y	NA	NA	Death

11.	[21]	M, 1.6	T-NHL			Y	Y	NA	NA	Death

12.	[21]	M, 13.7	Mixed centroblastic/ centrocytic NHL			Y	NA	NA	NA	Alive

13.	[21]	M, 7.2	B- NHL			N	NA	NA	NA	Death

14.	[22]	F, 4.6	B-cell NHL	Glioblastoma multiforme	right iliac fossa mass	N	N	CALM, neurofibromas	Chemotherapy	Alive

15.	[23]	M, 65	Diffuse mixed type, T cell type NHL		generalized lymphadenopathy	Y	NA	CALM, neurofibromas	Chemotherapy	Death

16.	[24]	F, 2**	Undifferentiated NHL			Y	Y	CALM, neurofibromas, pseudoarthrosis	NA	Death

17.	[25]	M, 3.3**	NHL			Y	Y	CALM	NA	Death

18.	[26]	M, 44	Cutaneous T-cell lymphoma	Astrocytoma	skin rash	NA	NA	CALM, neurofibromas, freckles	Phototherapy and topical steroid	Alive

19.	[27]	F, 77	DLBCL		left lower abdominal mass and weight loss	Y	NA	CALM, neurofibromas	Chemotherapy	Alive

20.	[28]	M, 2	T-cell NHL	Colorectal cancer	testicular and mediastinal mass	Y	NA	CALM, freckle	Chemotherapy and radiotherapy	Alive

21.	[29]	M, 72	Cutaneous T-cell lymphoma		skin rash	Y	NA	CALM, neurofibromas, freckles, Lisch nodules	NA	Alive

22.	[30]	M, 47	CNS lymphoma, B cell type		parieto-occipital lobe mass	NA	NA	Neurofibromas, Lisch nodules	NA	NA

23.	[31]	M, 9.4	T-cell lymphoma	Anaplastic astrocytoma	mediastinal mass	N	Y	CALM, freckles	Chemotherapy	Death

24.	[32]	M, 5***	Lymphoblastic lymphoma	Colorectal cancer		Y	Y	CALM, freckles	NA	Death

25.	[33]	F,50	DLBCL		shoulder mass	N	N	CALM, neurofibromas	Chemotherapy	Alive

26.	[34]	F, 6	Mediastinal T-cell lymphoblastic lymphoma	Gaucher's disease		NA	NA	NA	NA	NA

27.	Present case	M, 28	DLBCL		neck mass	N	N	CALM, neurofibromas, freckles, Lisch nodules	Chemotherapy	Alive

Abbreviations: CALM, café au lait macules; CNS, central nervous system; DLBCL, diffuse large B-cell lymphoma; F, female; M, male; N, No; NA, not available; NF, neurofibromatosis; NF1, neurofibromatosis type 1; NHL, non-Hodgkin's lymphoma; y, years; Y, Yes;

*Freckles include axillary or inguinal freckles

** Presence of MLH1 (human mutL homolog 1) mutation

***Presence of MSH6 (human mutS homolog 6) mutation

With respect to the symptoms of NF1 in the reported series, 20 cases had café au lait macules (CALM), 13 cases had neurofibromas, five cases had axillary or inguinal freckles, and three cases had Lisch nodules. These cases were typical of NF1 in which CALM, neurofibroma and skin-fold freckling are the main symptoms [36]. Most of the reported cases (17 cases) had a family history of neurofibromatosis. Twelve cases had family members with neurofibromatosis and malignancies. All cases were diagnosed with non-Hodgkin's lymphoma (NHL). The majority of cases were young with a mean age of 23 years (range 1.1 to 77 years; median 9.4 years) at lymphoma diagnosis and two-thirds of the cases were boys and men. The lymphoma subtype was predominantly T-cell (13 of 25 cases), especially T-lymphoblastic lymphoma (five cases) and cutaneous T cell lymphoma (two cases). Nine cases had B-cell lymphoma and five cases had unclassified lymphoma. Six cases presented with a mediastinal mass and others presented with a head and neck mass (three cases), an abdominal mass (three cases), lymphadenopathy (two cases), skin rash (two cases), a localized brain lesion (one case), and a shoulder mass (one case). No NF1 patients in prior series were reported to present with Horner's syndrome. Other concomitant malignancies identified were brain tumors (four cases), colorectal cancer (two cases), pheochromocytoma (two cases), and acute lymphoblastic leukemia (ALL) (two cases). One case developed ALL prior to NHL [21] and the remaining case developed ALL 15 months after diagnosis of NHL [7]. Additional disorders found among the 26 cases were Gardner syndrome (one case) and Gaucher's disease (one case). Thirteen cases received standard chemotherapy while six cases received radiotherapy. None of the NF1 cases received stem cell transplantation. Ten patients (37%) responded to therapy and survived. Our patient also responded well to eight cycles of CHOP chemotherapy with resolution of his Horner's syndrome and disappearance of all tumor masses. Immunotherapy such as rituximab was not given in this particular patient due to limited availability of health insurance coverage for such costly drugs in the country.

Although the central nervous system involvement of malignant lymphoma is well documented, the interruption of the sympathetic pathway causing Horner's syndrome is an unusual manifestation of malignant lymphoma [37]. In our current review of the literature, there was only one described case of Burkitt's lymphoma presenting with toothache, paresthesia and Horner's syndrome [13,37]. Hodgkin's disease was also infrequently reported [7,16-34]. No cases of DLBCL as the cause of Horner's syndrome have been described in the literature. The mechanism of Horner's syndrome in this patient could be explained by the aggressive nature of DLBCL creating a huge mass in the anterior and middle mediastinum which encased his aortic arch, subclavian artery, left jugular vein, and left pulmonary artery, and extended to his left neck along the carotid vessels. The tumor mass could potentially affect the oculosympathetic pathway that normally enters the thorax, crosses the lung apex, and subsequently runs along the carotid vessels to the eyes.

The direct association between NF1 and T-cell lymphoma in most previously reported cases or between NF1 and DLBCL in this present case is unclear. The NF1 gene is a tumor suppressor gene and multiple key pathways are potentially involved in the development of cancer in NF1 cases such as RAS/mitogen-activated protein kinase (MAPK) and AKT/mammalian target of rapamycin (mTOR) [38,39]. Germline mutations of the NF1 generally result in decreased intracellular neurofibromin protein levels and lead to increased RAS signaling to its downstream effectors [38]. Mutations in the DNA mismatch repair genes, such as MLH1 (human mutL homolog 1) [24,25] and MSH6 (human mutS homolog 6), have been reported in NF1 cases with malignant lymphoma (as shown in Table 1), early-onset CNS tumors, and colorectal cancer [31]. Lymphoma potentially occurs in NF1 patients through a series of proto-oncogene activation and mismatch repair gene mutations although the precise pathogenetic mechanism needs to be further explored.

## Conclusion

DLBCL has never been shown to manifest initially as Horner's syndrome. To the best of our knowledge, our case represents the first case ever of Horner's syndrome that occurred as a result of oculosympathetic interruption by DLBCL. This case reveals a rare association between NF1 and DLBCL in contrast to other more common non-hematologic malignancies that frequently occurred in NF1 cases. Molecular mechanisms required for the initiation and propagation of lymphoma in NF1 cases should be determined to answer why and how only a very few NF1 cases are at risk of lymphoma development in their lifetimes.

### Consent

Written informed consent was obtained from the patient for publication of this case report and any accompanying images. A copy of the written consent is available for review by the Editor-in-Chief of this journal.

## Competing interests

The authors declare that they have no competing interests.

## Authors' contributions

SL performed data collection and analysis and drafting of the manuscript. CUA was SL's major advisor who was responsible for the critical revision of the manuscript. Both authors read and approved the final manuscript.
